# The integration of plasma non-target metabolomics and lipidomics analysis for the discovery of global developmental delay/intellectual disability biomarkers

**DOI:** 10.3389/fncel.2026.1688339

**Published:** 2026-02-04

**Authors:** Baiyu Chen, Shimeng Chen, Juan Xiong, Miriam Kessi, Jing Peng, Fei Yin, Fang He

**Affiliations:** 1 Department of Pediatrics, Affiliated Hospital of Inner Mongolia Medical University, Hohhot, China; 2 Department of Pediatrics, Xiangya Hospital, Central South University, Changsha, China; 3 Hunan Intellectual and Developmental Disabilities Research Center, Changsha, China

**Keywords:** biomarkers, global developmental delay, intellectual disability, lipidomics, metabolomics

## Abstract

**Background:**

This study aimed to identify metabolic signatures and potential biomarkers for global developmental delay (GDD) and intellectual disability (ID) using plasma metabolomics and lipidomics. The research sought to evaluate the feasibility of these methods for early identification and to explore the underlying metabolic pathways associated with GDD/ID.

**Methods:**

A liquid chromatography coupled with tandem mass spectrometry (LC–MS/MS)-based method integrated with multivariate data analysis was employed to comprehensively characterize plasma metabolomics and lipidomics profiles in children diagnosed with GDD/ID compared to typically developing (TD) children. The study focused on identifying distinct metabolites and lipids that could differentiate GDD/ID from TD children.

**Results:**

The analysis revealed that a combination of 11 metabolites and lipids could effectively discriminate between GDD/ID and TD children. Receiver operating characteristic (ROC) analysis identified several potential biomarkers for GDD/ID. In positive ion mode, glycerophosphocholine (AUC = 0.899) and sphinganine (AUC = 0.859) showed diagnostic potential. Negative ion mode analysis revealed five biomarkers, notably 2-ketohexanoic acid (AUC = 0.912) and N-acetyl-L-aspartic acid (AUC = 0.870). Lipidomics analysis highlighted two high-performance biomarkers: diacylglycerol (DAG) (16:0/16:0) (AUC = 0.956) and DAG (16:0/18:0) (AUC = 0.949). Key metabolic pathways associated with GDD/ID included D-glutamine, D-glutamate, alanine, aspartate, glutamate, sphingolipid, histidine, arginine, and proline metabolisms. Furthermore, lysine metabolic pathways, including degradation and biosynthesis, as well as aminoacyl-tRNA biosynthesis, were implicated in GDD/ID pathogenesis.

**Conclusion:**

This study identified putative biomarkers and metabolic pathways associated with GDD/ID, highlighting the potential of combined plasma metabolomics and lipidomics for early screening of GDD/ID and providing tentative insights into its pathophysiology. The biomarkers show strong diagnostic performance as screening tools, but future studies are needed to validate their prognostic value and clinical utility in multi-center cohorts.

## 1 Introduction

Global developmental delay (GDD) is defined as a failure to achieve developmental milestones in children aged 5 years or younger. Intellectual disability (ID) encompasses general mental ability problems that affect both intellectual and adaptive functioning before the age of 18 years (Vasudevan and Suri, 2017). The etiologic investigations of GDD and ID exhibit significant overlap, with shared pathogenic mechanisms and risk factors. GDD/ID affect up to 3% of the pediatric population and are associated with substantial comorbidities, emotional burden, and high lifetime costs (Bélanger and Caron, 2018; van Hoytema Konijnenburg et al., 2021). Therefore, early identification of GDD/ID is essential for timely diagnosis and provision of treatment and rehabilitation services.

The multifactorial etiology of GDD/ID necessitates the identification of reliable biomarkers, which may elucidate pathogenic mechanisms and inform targeted therapeutic strategies. At present, genetic metabolic diseases account for 1–5% of the etiology of GDD/ID (van Hoytema Konijnenburg et al., 2021; van Karnebeek, 2014). The spectrum of metabolic disorders implicated in GDD/ID encompasses multiple biochemical pathways, such as amino acid metabolism disorders (Lichter-Konecki and Vockley, 2019; Burrage et al., 2014; Yoo et al., 2020; Lim et al., 2020), urea cycle disorders (Matsumoto et al., 2019), organic acid metabolism disorders (Gabbi et al., 2017), lysosomal storage diseases (Boustany, 2013), peroxisomal disorders (Berger et al., 2016), mitochondrial diseases (Flippo and Strack, 2017), creatine deficiency syndrome (Sharer et al., 2017), glucose transport protein deficiency (Daci et al., 2018), abnormal metabolism of purines and pyrimidines (García-Cazorla et al., 2009), cholesterol synthesis disorders (DeBarber et al., 2011), and congenital glycosylation abnormalities (Reily et al., 2019). Altogether, these abnormalities underscore the complex etiology linking metabolic dysregulation to GDD/ID pathogenesis, leading to the exploration of models for multivariate analysis.

In recent years, the rapid development of high-throughput methods has enabled researchers to better understand the pathogenesis and complex metabolic disorders of neurodevelopmental disorders, including GDD/ID. As emerging fields for identifying and characterizing biomarkers, non-target metabolomics and lipidomics have made progress in the diagnosis of neurodevelopmental disorders in children (Chen et al., 2019). Metabolomics approaches are being used to investigate the etiologies and pathogenesis of various diseases, as well as to monitor the efficacy of treatments used to improve metabolism (Wishart, 2016). Non-target metabolomics primarily aims to detect as many low molecular weight substances as possible in the body and to analyze the metabolic spectrum of cells, tissues, organs, or biological fluids under the influence of different factors. Lipidomics is one of the fastest-growing fields in systems biology research. It is a new member of omics research represented by metabolomics and proteomics. Lipidomics can qualitatively and quantitatively detect the levels of lipid molecules in organisms. Like metabolomics, it can also be applied to explore the pathogenesis of diseases, early identification of diseases, assessment of disease prognosis, and evaluation of drug efficacy (Züllig et al., 2020; Chen et al., 2019). Considering the uniqueness and complexity of lipidomes in the nervous system, the application of lipidomics is extremely important (Han et al., 2012).

While metabolomic profiling has emerged as a promising approach in neurodevelopmental research, with well-characterized biomarkers identified in autism spectrum disorders (ASD), including dysregulations in microbial metabolites, niacin metabolism, mitochondria-related metabolites, and amino acid metabolites (Chen et al., 2019), comprehensive metabolomic characterization of GDD/ID remains critically underexplored. This study aimed to identify metabolic signatures and potential biomarkers for diagnosis, as well as possible etiologies of GDD/ID. Thus, we used LC–MS/MS-based metabolomics and lipidomics to identify different metabolites and lipids. The findings of this study will help to better understand the molecular mechanisms, improve early diagnosis, and provide guidance in the provision of precise treatment for children with GDD/ID.

## 2 Materials and methods

### 2.1 Participants

Sixty participants were recruited, comprising 30 pediatric patients with unexplained GDD/ID and 30 TD children in the control group. All participants were recruited from the Children’s Health Clinic at Xiangya Hospital, Central South University, and included Han Chinese individuals with comparable dietary patterns and shared geographical residency to minimize potential confounding factors. Parents completed a structured dietary questionnaire to confirm that their children maintained a regular, balanced diet consistent with local eating patterns, with no history of malnutrition, restrictive diets, or nutrient deficiencies. Furthermore, none of the participants had used chronic medications (e.g., anticonvulsants or psychotropic drugs) or nutritional supplements (including vitamins, minerals, or herbal products) for at least 3 months prior to blood sampling, thereby reducing potential confounding from exogenous interventions. This study was approved by the Institutional Ethics Committee of Xiangya Hospital, Central South University. Both informed and written consents were obtained from the parents and/or legal guardians for study participation.

Inclusion criteria for GDD/ID were as defined by the Diagnostic and Statistical Manual of Mental Disorders, Fifth Edition (DSM-V). Global Developmental Delay (GDD) is defined as a significant delay (≥2 standard deviations below the age-appropriate mean) in two or more developmental domains, including gross motor, fine motor, speech/language, cognitive, social–emotional, and adaptive functioning, in children aged ≤5 years. Diagnosis was confirmed through three steps: (1) clinical evaluation by two senior pediatric neurologists with expertise in neurodevelopmental disorders; (2) standardized developmental assessment using the Bayley Scales of Infant and Toddler Development, 3rd Edition (BSID-III), with composite scores ≤70 in the affected domains; (3) exclusion of acquired or structural causes, such as perinatal hypoxia, intracranial hemorrhage, pathological jaundice, and central nervous system infection, via medical record review and neuroimaging (when indicated). Intellectual Disability (ID) is defined as deficits in general intellectual functioning and adaptive functioning that manifest before 18 years of age. General intellectual functioning was assessed using the Wechsler Intelligence Scale for Children, 4th Edition (WISC-IV), with an IQ score ≤70. Adaptive functioning was evaluated via the Vineland Adaptive Behavior Scales, 3rd Edition (VABS-III), with a composite score ≤70. For children >5 years in the cohort, ID was diagnosed only if GDD had persisted beyond the preschool period, with no evidence of regression or new-onset neurological insult. Exclusion criteria included: (1) presence of other diseases that may affect the development of the nervous system (e.g., intrauterine distress, history of hypoxia at birth, pathological jaundice, intracranial bleeding, perinatal infection, central nervous system infection, poisoning, and trauma history); (2) presence of chromosomal abnormalities or monogenic or polygenic diseases that can cause GDD/ID. All participants were examined by senior pediatric neurologists. The detailed demographic profiles of the participants are provided in Supplementary Table S1.

### 2.2 Sample preparation

All blood samples were collected between 7:00 and 9:00 a.m. after an 8–12-h overnight fast to avoid metabolic variability induced by recent food intake. Participants were instructed to abstain from all foods and beverages (except plain water) during the fasting period, and parents confirmed adherence to the fasting protocol prior to sampling. Blood samples (2–3 mL) from participants were collected before breakfast using vacuum anticoagulant tubes, and were centrifuged at 3,000 rpm at 4 °C for 10 min. The plasma supernatants were harvested into 1.5 mL freezing tubes, snap-frozen in liquid nitrogen, and stored at −80 °C until used for metabolomic and lipidomic assays.

For plasma metabolomics, 100 μL of each sample was transferred to an Eppendorf (EP) tube. About 400 μL of extract solution (acetonitrile: methanol = 1:1, containing isotopically labeled internal standard mixture) was added to each tube. The tubes were vortexed for 30 s, sonicated for 10 min in an ice–water bath, and incubated for 1 h at −40 °C to precipitate proteins. The tubes were then centrifuged at 12000 rpm (RCF = 13,800 (×g), R = 8.6 cm) for 15 min at 4 °C. The resulting supernatants were transferred to a fresh glass vial for analysis. For plasma lipidomics, 10 μL of each sample was mixed with 190 μL of water, and then 480 μL of extract solution (methyl tert-butyl ether (MTBE): methanol = 5:1) containing the internal standard was added. The glass vials were vortexed for 60 s and then sonicated for 10 min in an ice–water bath. The tubes containing samples were centrifuged at 3,000 rpm for 15 min at 4 °C. About 250 μL of supernatants were transferred to fresh tubes, and an additional 250 μL of MTBE was added to the remaining samples. The tubes were then vortexed, sonicated, and centrifuged again as before, and another 250 μL of supernatants were collected. This step was repeated once. All supernatants were combined and dried in a vacuum concentrator at 37 °C. The dried samples were then reconstituted in 100 μL of resuspension buffer (Dichloromethane (DCM): Methanol (MeOH): H_2_O = 60:30:4.5). The samples were vortexed for 30 s and sonicated for 10 min in an ice–water bath. The solutions were then centrifuged at 12,000 rpm for 15 min at 4 °C, and 30 μL of supernatants were transferred to fresh glass vials for LC/MS analysis. The quality control (QC) sample was prepared by mixing equal aliquots of the supernatants from all samples.

### 2.3 Data preprocessing for metabolomics and lipidomics

Mixed omics data (metabolites + lipids) in Supplementary Tables S3, S4 were standardized using R software (v4.2.1) with the metaboAnalystR and sva packages. The preprocessing workflow was identical for both tables, as detailed below: (1) Normalization: For each Supplementary Tables S3, S4, calculate the median signal intensity of “all detected features (metabolites + lipids)” in each sample. Scale the intensity values of all metabolites and lipids within a sample using the ratio of the table’s “global reference median” to the sample’s median, eliminating non-biological variability. (2) Batch Correction: Data in both tables were acquired in two batches, so the ComBat-seq algorithm was used for batch correction—“batch” was treated as a random variable, and “biological group” was included as a covariate to ensure true signals of metabolites and lipids were not masked. After correction, the CV of QC samples was < 15%, with no differences in correction efficacy between metabolites and lipids. (3) Missing Value Handling: The proportion of missing values for metabolites and lipids in both tables was <3%, and all missing values were imputed using the k-nearest neighbors (k-NN) method (*k* = 5). A threshold of “excluding features with >50% missing values” was pre-defined, but no metabolites or lipids in either table met this threshold, so all features were retained.

### 2.4 LC–MS/MS analysis

For plasma metabolomics, LC–MS/MS analyses were performed using a UHPLC system (Vanquish, Thermo Fisher Scientific) coupled with a Q Exactive HFX Orbitrap mass spectrometer (Thermo Fisher Scientific). Chromatographic separation was achieved on a UPLC BEH Amide column (2.1 mm × 100 mm, 1.7 μm; Waters) maintained at 40 °C. The mobile phase consisted of solvent A (25 mmol/L ammonium acetate + 25 mmol/L ammonia hydroxide in water, pH 9.75) and solvent B (acetonitrile). The gradient elution program was as follows: 0–2 min, 95% B; 2–10 min, 95–60% B; 10–12 min, 60–40% B; 12–14 min, 40% B; 14–14.1 min, 40–95% B; 14.1–18 min, 95% B. The flow rate was 0.3 mL/min, the auto-sampler temperature was 4 °C, and the injection volume was 2 μL. Mass spectrometry was operated in both positive and negative electrospray ionization (ESI) modes. The ESI source parameters were: sheath gas flow rate = 30 Arb, auxillary gas flow rate = 25 Arb, capillary temperature = 350 °C, full MS resolution = 60,000, MS/MS resolution = 7,500, collision energy = 10/30/60 eV (normalized collision energy, NCE) for data-dependent acquisition. The spray voltage was set to 3.6 kV (positive mode) or −3.2 kV (negative mode). Data were acquired in information-dependent acquisition (IDA) mode using Xcalibur software (v4.3, Thermo Fisher Scientific), with dynamic exclusion of precursor ions for 10 s to avoid redundant fragmentation.

For plasma lipidomics, UHPLC separation was conducted on a SCIEX ExionLC series system equipped with a Kinetex C18 column (2.1 mm × 100 mm, 1.7 μm; Phenomenex) maintained at 40 °C. The mobile phase A consisted of 40% water and 60% acetonitrile containing 10 mmol/L ammonium formate, while mobile phase B comprised 10% acetonitrile and 90% isopropanol containing 10 mmol/L ammonium formate. The gradient elution program was as follows: 0–2 min, 30% B; 2–10 min, 30–60% B; 10–14 min, 60–98% B; 14–18 min, 98% B; 18–18.1 min, 98–30% B; 18.1–22 min, 30% B. The flow rate was 0.35 mL/min, the auto-sampler temperature was set at 6 °C, and the injection volume was 2 μL. Mass spectrometry was performed on an AB Sciex QTrap 6,500 + system in both positive and negative ESI modes. The ion source parameters were as follows: ion spray voltage = +5,500/−4,500 V, curtain gas = 40 psi, source temperature = 350 °C, ion source gas 1 = 50 psi, ion source gas 2 = 50 psi, and declustering potential (DP) = ±80 V. Lipid identification and quantification were performed using Biobud-v2.07 software (BiotreeDB, Shanghai, China) with a built-in lipid library, and quantification was based on internal standard calibration (corresponding class-specific lipid internal standards).

### 2.5 Raw data processing

For plasma metabolomics, raw MS data (.raw files) were converted to mzXML format using ProteoWizard (v3.0.21246). Peak detection, alignment, and integration were performed using an in-house R script based on the XCMS package (v3.18.1). The parameters for peak picking were as follows: centWave method with ppm = 15, peakwidth = c(5, 60) s, and snthresh = 3. Peak alignment was conducted using the obiwarp method (profStep = 1), and peak filling was performed with the fillPeaks function. Metabolite annotation was achieved by matching MS/MS spectra to the in-house BiotreeDB library (v2.1) with a similarity threshold of 0.3 (based on MS/MS fragment ion matching and retention time consistency). Only metabolites with annotation scores ≥0.3 and present in ≥80% of samples in at least one group were retained for further analysis.

For plasma lipidomics, raw data were processed using Biobud-v2.07 software. Lipid identification was confirmed by matching precursor ion mass (±5 ppm) and MS/MS fragment patterns to the Biotree lipid database. Quantification was performed using the internal standard method: the absolute concentration of each lipid was calculated by comparing its peak area to the peak area of the corresponding class-specific internal standard (e.g., phosphatidylcholine internal standard for PC lipids) with a known concentration. Lipids with a coefficient of variation (CV) > 20% in quality control (QC) samples were excluded to ensure data reliability.

### 2.6 Modeling and statistical analysis

Variables conforming to the normal distribution were expressed as mean ± standard deviation, and measurement data not conforming to the normal distribution were expressed as median or quartiles. A two-sample independent *t*-test was used for comparison. Categorical data were summarized in the form of frequencies and proportions and analyzed using the chi-square test or Fisher’s exact test where applicable. The level of significance was set at *p* ≤ 0.05. Prior to statistical analysis, metabolomic and lipidomic data were preprocessed as described in Section 2.3 (Data Preprocessing for Metabolomics and Lipidomics). For univariate analysis, two-sample independent *t*-tests were used to compare metabolite/lipid levels between the GDD/ID and TD groups, with *p* < 0.05 considered statistically significant. Fold change (FC) was calculated as the ratio of the mean intensity in the GDD/ID group to that in the TD group, with FC ≥ 1.2 or ≤ 0.8 indicating a meaningful change.

For multivariate analysis, unsupervised principal component analysis (PCA) was performed using the prcomp function in R (v4.2.1) to visualize global data distribution and detect outliers. Outliers were identified using the Mahalanobis distance method (*p* < 0.01) and excluded from further analysis. Supervised orthogonal partial least-squares discriminant analysis (OPLS-DA) was conducted using the ropls package (v1.30.0) to maximize the separation between groups. The model performance was evaluated by R^2^X, R^2^Y, and Q^2^ values, with R^2^Y and *Q*^2^ > 0.5 indicating good model fit. We performed a test with 200 permutations to assess the validity of the discriminant models and avoid overfitting, with a Q^2^ intercept <0 confirming no overfitting.

Differential metabolites/lipids were defined as those meeting VIP > 1.0 (from the OPLS-DA model), *p* < 0.05 (*t*-test), and FC ≥ 1.2 or ≤0.8. Multiple-testing correction was applied using the Benjamini–Hochberg procedure to calculate the false discovery rate (FDR), with FDR < 0.05 as the final threshold for significant differences. To address the risk of false positives from “mixed features (metabolites + lipids)” in Supplementary Tables S3, S4, independent multiple-testing corrections were performed for each table: (1) The Benjamini–Hochberg procedure was used to calculate FDR-corrected *p*-values, with FDR < 0.05 as the statistical threshold for differential features; (2) In Supplementary Tables S3, S4, the “Q-VALUE” column represents unified FDR-corrected *p*-values for metabolites and lipids, and all differential features meet FDR < 0.05; (3) When screening differential features, in addition to FDR < 0.05, “VIP > 1.0” and “FC ≥ 1.2 or ≤0.8” were also applied, with identical criteria for metabolites and lipids to ensure result consistency.

Metabolites with VIP > 1 and *p* < 0.05 were analyzed using a heatmap and were employed for pathway enrichment analysis. Pathway enrichment analysis was performed using MetaboAnalyst 5.0^1^ with the KEGG (Kyoto Encyclopedia of Genes and Genomes) human metabolic pathway database. Enriched pathways were identified using hypergeometric tests, and the results were visualized as bubble plots, with −log10(*p*-value) on the y-axis and pathway impact value (from topology analysis) on the x-axis. Pearson correlation analysis was conducted between differential metabolites/lipids using the cor function in R, with correlation coefficients (r) visualized as heatmaps (pheatmap package, v1.0.12). Only significant correlations (*p* < 0.05) were included in the heatmaps. MetaboAnalyst 5.0, the KEGG database, the Metlin database, and the HMDB database were used to analyze relevant data and metabolic pathways.

#### 2.6.1 Power and sample size consideration

Given the exploratory nature of this study, a formal *a priori* power calculation was not performed. However, a post-hoc power analysis was conducted based on the observed effect sizes of key differential metabolites (AUC > 0.85). Using G*Power 3.1 with an alpha of 0.05 and effect size derived from ROC analysis, the achieved power exceeded 80% for the primary biomarkers, indicating that the sample size was adequate to detect statistically significant differences in these selected metabolites.

#### 2.6.2 Model validation

To minimize the risk of overfitting, all supervised models (OPLS-DA) were validated using 200 permutation tests. A model was considered valid if the intercept of the permutation plot showed $Q_{2}<0$, indicating no overfitting.

#### 2.6.3 Covariate adjustment rationale

Fasting conditions, nutritional status, and pharmacologic exposures were standardized across the GDD/ID and TD groups (with no significant between-group differences in these variables), so these factors were not included as covariates in the statistical models. This rationale is explicitly stated to ensure transparency.

### 2.7 Screening of candidate diagnostic biomarker

Two machine learning algorithms, the support vector machine recursive feature elimination (SVM-RFE) and least absolute shrinkage and selection operator (LASSO), were used in this study to screen for GDD/ID-related biomarkers. SVM-RFE represents a widely used supervised machine-learning protocol for classification and regression that is applied using the “e1071” package. LASSO was performed using the “glmnet” package in R and represents a regression analysis algorithm that applies regularization for variable selection. We analyzed overlapping metabolites from the two algorithms for diagnostic biomarkers of GDD/ID.

The combination of SVM-RFE and LASSO algorithms was selected for biomarker screening based on their complementary strengths in handling high-dimensional omics data. SVM-RFE (support vector machine recursive feature elimination) is a supervised machine learning algorithm that excels at capturing non-linear relationships and interactions between features, which is critical for metabolomic/lipidomic data characterized by high dimensionality and complex biological correlations. It works by training an SVM model, ranking features based on their contribution to the model (using the weight vector of the SVM hyperplane), and recursively eliminating the least important features until an optimal feature subset is obtained. This process effectively retains features with strong discriminative power even in the presence of redundant or noisy data.

## 3 Results

### 3.1 Raw data preprocessing results

Preprocessing of non-target metabolomics raw data. The ionization source of the QE platform was electrospray; therefore, two ionization methods were used to achieve higher metabolite coverage and better detection. The methodsused included positive and negative ion modes. The original data included 9 quality control (QC) samples and 60 experimental samples. A total of 5,964 peaks were extracted from the data in positive ion mode and 4,956 from the data in negative ion mode. To reduce the impact of detection system errors and better highlight the biological significance of the results, we carried out a series of preparations and data curation, mainly including deviation value filtering, missing value filtering, missing value filling, and data standardization. After pretreatment, a total of 4,228 peaks in positive mode and 3,728 peaks in negative mode remained.

Preprocessing of full quantitative lipidomics raw data. The raw data included 8 QC samples and 60 experimental samples, from which 722 peaks were extracted, and 722 peaks remained after pretreatment.

### 3.2 Multivariate statistical analysis results

As an unsupervised model, PCA can reveal the internal structure of data, identify differences between samples in multi-dimensional space, and provide a preliminary understanding of the grouping among groups from an overall perspective. The PCA model parameters for non-target metabolomics and lipidomics data are shown in Supplementary Table S2. From the score scatter plot of the PCA model, it can be seen that the abscissa PC [1] and ordinate PC [2] represent the first and second principal component scores, respectively. Each scatter point represents one sample, with the color and shape indicating different groups. The closer the distribution of sample points, the more similar the types and contents of metabolites in the samples. The samples were predominantly within the 95% confidence interval (CI) (Supplementary Figures S1A–C).

We further used the supervised OPLS-DA multivariate statistical method to filter out orthogonal variables that were not related to the categorical variables, and then analyzed the non-orthogonal and orthogonal variables separately to maximize the reliable differences between the two groups. The OPLS-DA model parameters for non-target metabolomics and lipidomics data are shown in Supplementary Table S2. The score scatter plot of the OPLS-DA model showed that the two groups could be well distinguished. The samples were predominantly within the 95% CI (Supplementary Figures S1D–F).

Finally, we performed an additional evaluation of the OPLS-DA model through permutation testing. In the permutation test of the OPLS-DA model, Q_2_<0 indicated that the model was successfully constructed and that no overfitting occurred (Supplementary Figures S1G–I).

### 3.3 Metabolomics and lipidomics differential metabolites between GDD/ID and TD groups

The variables of *p* < 0.05 and VIP > 1.0 were selected as candidate differential metabolites. The differential metabolites of the positive and negative ion modes in the GDD/ID and TD groups are listed in Supplementary Table S3 (POS and NEG). The differential lipids in the GDD/ID and TD groups are listed in Supplementary Table S3 (LIPIDS). All features in Supplementary Table S3 (including metabolites and lipids) were preprocessed via probabilistic quotient normalization (PQN), ComBat-seq batch correction, and k-nearest neighbors (k-NN) missing value imputation. The “Q-VALUE” column in the table represents false discovery rate (FDR)-corrected *p*-values (Benjamini–Hochberg procedure), with all 108 differential metabolites and 48 lipids meeting the threshold of FDR < 0.05. According to this criterion, 108 differential metabolites and 48 lipids were identified between the GDD/ID and TD groups. To visualize the general distribution, we displayed the results of the differential metabolite screening as volcano plots (Supplementary Figures S2A–C). The differential metabolites identified in metabolomics mainly included lipid and lipid-like molecules, organic acids and derivatives, organic nitrogen compounds, organoheterocyclic compounds, organic oxygen compounds, nucleosides, nucleotides and analogs, hydrocarbons, phenylpropanoids, polyketides, and benzenoids (Supplementary Figures S2D,E). The differential lipids identified in lipidomics mainly included diacylglycerols (DAG), free fatty acids (FFA), lysophosphatidylcholines (LPC), lysophosphatidylethanolamines (LPE), phosphatidylcholines (PC), phosphatidylethanolamines (PE), and triacylglycerols (TAG) (Supplementary Figure S2F).

### 3.4 Correlation analysis of differential metabolites between non-target metabolomics and lipidomics groups

Metabolite correlation often reveals the coordination of changes between metabolites. A positive correlation indicates that the change trend is the same as that of a certain class of metabolites, while a negative correlation indicates the opposite. To gain insight into the relationship between differential metabolites, we performed a Pearson correlation analysis (Supplementary Figure S3). The degree of correlation between two variables was expressed by the correlation coefficient r. For a positive correlation, the *r* value ranged from 0 to 1. For a negative correlation, the *r* value ranged from −1 to 0. The closer the absolute value of r was to 1, the stronger the degree of association between the two variables; conversely, the closer the absolute value of r was to 0, the weaker the association (Supplementary Table S4). All features in Supplementary Table S4 (including metabolites and lipids) share the same preprocessing workflow as Supplementary Table S3 (PQN normalization + ComBat-seq batch correction + k-NN imputation). The “Q-VALUE” column in the table represents FDR-corrected *p*-values (Benjamini–Hochberg procedure), with all differential features meeting FDR < 0.05.

### 3.5 Pathway enrichment analysis of differential metabolites between non-target metabolomics and lipidomics data groups

**Figure 1:**
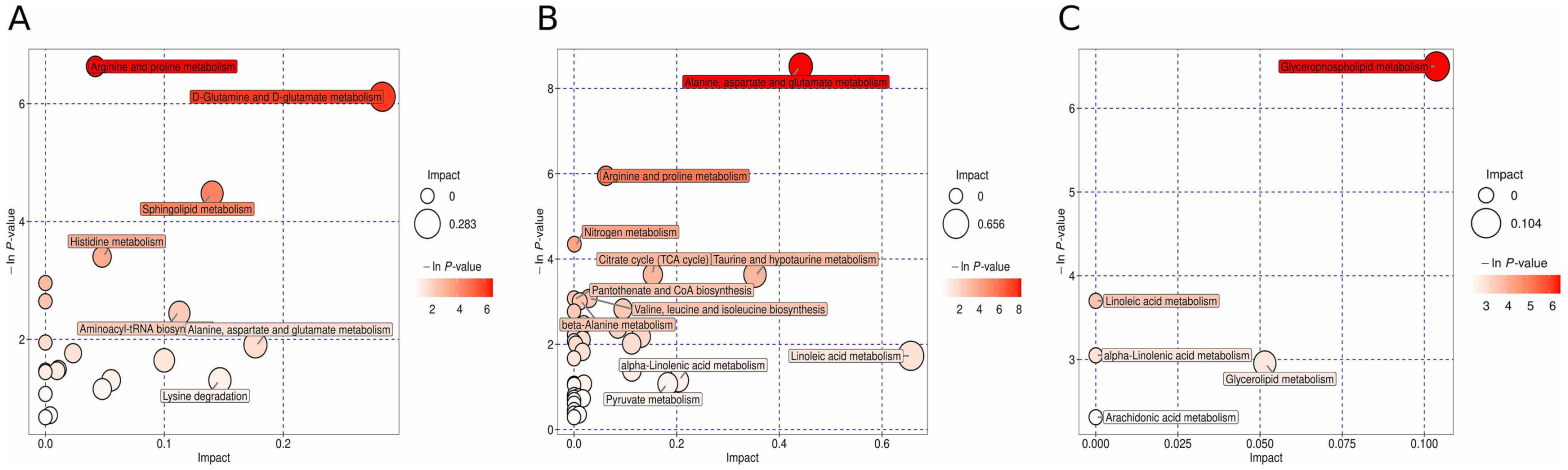
Pathway enrichment analysis of differentially expressed metabolites and lipids between GDD/ID and TD groups. Bubble diagrams showing pathways of differential metabolites in the positive ion mode **(A)**, negative ion mode **(B)**, and lipids **(C)** between GDD/ID patients and TD children. Each bubble in the diagram represents a metabolic pathway. The position of the bubble on the x-axis and its size represent the influence of the pathway in the topology analysis. The position of the bubble on the y-axis and its color represent the *p*-value of the enrichment analysis [the negative natural logarithm is taken, that is, −ln (*p*)].

The selected differential metabolites were matched in KEGG, PubChem, and other metabolite databases. They were further searched in the pathway database of the corresponding species, *Homo sapiens* (human), for metabolic pathway analysis. The results are shown in a bubble diagram. We chose the dark-colored large bubbles (highly enriched pathways) as the focus of subsequent studies. For non-target metabolomics data, the metabolic pathways with significant enrichment of differential metabolites in the positive ion mode included D-glutamine, D-glutamate, alanine, aspartate, glutamate, histidine, arginine, sphingolipid, and proline metabolisms. Moreover, lysine degradation, aminoacyl biosynthesis, and lysine biosynthesis were included (Figure 1A). The metabolic pathways with significant enrichment of differential metabolites in the negative ion mode included linoleic acid, alanine, aspartate, glutamate, taurine, hypotaurine, the citric acid cycle, arginine, and proline metabolisms. In addition, valine, leucine, and isoleucine biosynthesis, β-alanine and nitrogen metabolisms, as well as pantothenic acid and coenzyme A biosynthesis were included (Figure 1B). For lipidomics data, the metabolic pathways with significant enrichment of differential metabolites included glycerolipid and glycerolipid metabolisms (Figure 1C).

### 3.6 Identification and verification of diagnostic biomarkers

**Figure 2:**
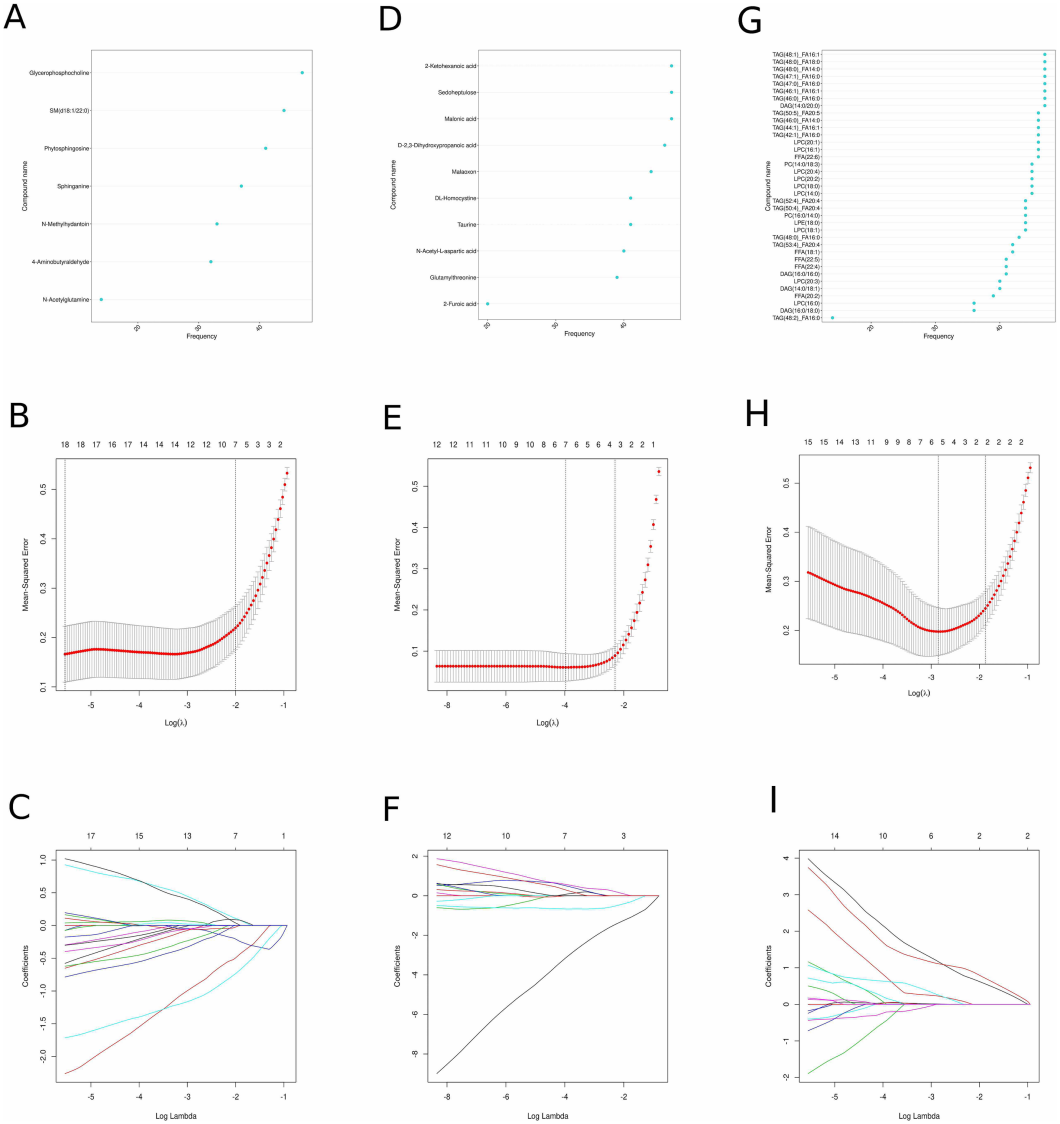
Screening characteristics related to differential metabolites via a comprehensive strategy. Characteristics related to differential metabolites **(A)**. Positive ion mode metabolites, **(D)** negative ion mode metabolites, and **(G)** lipids were screened by the SVM-RFE. **(B,C)** Positive ion mode metabolites, **(E,F)** negative ion mode metabolites, and **(H,I)** lipids were screened by the LASSO algorithm.

**Figure 3:**
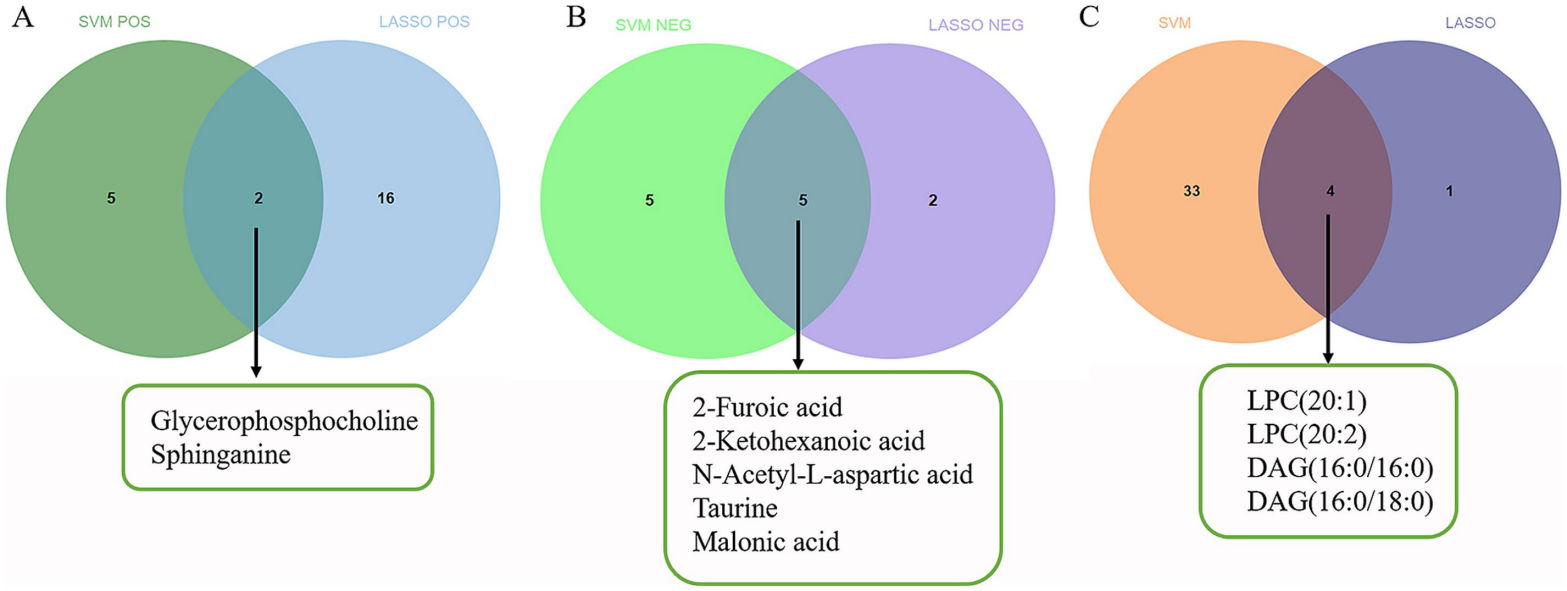
Venn diagrams of variables screened by SVM-RFE and LASSO algorithms. **(A)** The Venn diagram shows the intersection of differential metabolites in the positive ion mode obtained by SVM-RFE and LASSO algorithms. **(B)** The Venn diagram shows the intersection of differential metabolites in the negative ion mode obtained by SVM-RFE and LASSO algorithms. **(C)** The Venn diagram shows the intersection of differential lipids obtained by SVM-RFE and LASSO algorithms.

**Figure 4:**
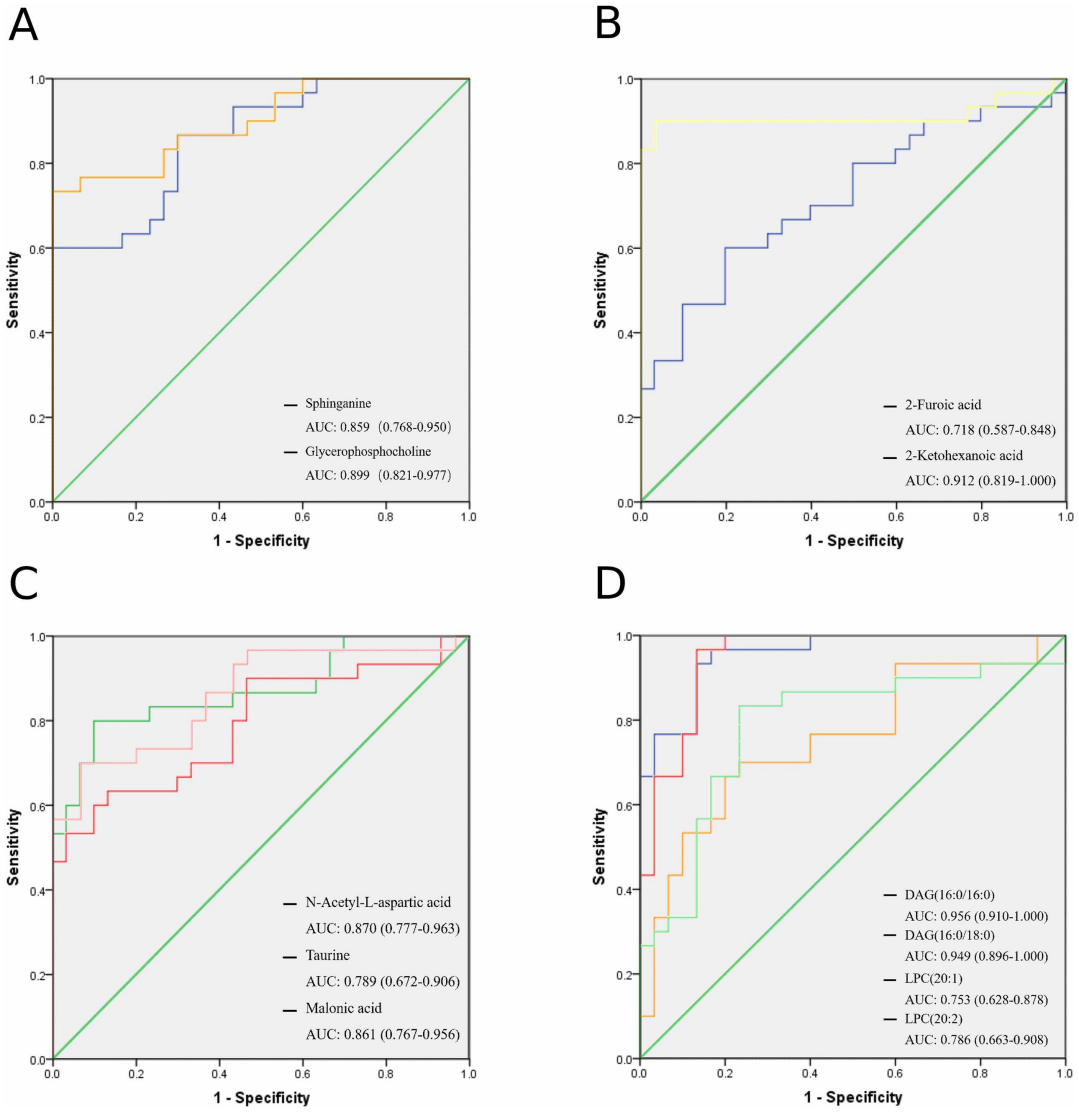
ROC curve analysis of the diagnostic value of differential metabolites. **(A)** ROC curve analysis of the diagnostic value of positive ion mode metabolites. **(B,C)** ROC curve analysis of the diagnostic value of negative ion mode metabolites. **(D)** ROC curve analysis of the diagnostic value of lipids.

We used two different algorithms (SVM-RFE and LASSO) to screen candidate diagnostic biomarkers. For non-target metabolomics data, SVM-RFE identified 7 features in positive ion mode and 10 features in negative ion mode (Figures 2A,D), while LASSO identified 18 differential metabolites in positive ion mode and 7 in negative ion mode (Figures 2B,C,E,F). For lipidomics data, SVM-RFE identified 37 potential lipid biomarkers, and LASSO identified 5 (Figures 2G,H,I). Through the intersection of the two algorithms, a total of 11 putative biomarkers were finally selected, including 2 metabolites from positive ion mode (glycerophosphocholine, sphinganine), 5 metabolites from negative ion mode (2-furoic acid, 2-ketohexanoic acid, N-acetyl-L-aspartic acid, taurine, malonic acid), and 4 lipid species [LPC (20:1), LPC (20:2), DAG (16:0/16:0), DAG (16:0/18:0)] (Figures 3A–C). These biomarkers are also visualized in Figure 4 (relative peak area comparisons between GDD/ID and TD groups), with detailed diagnostic performance metrics provided in Section 3.7 and Supplementary Table S5.

### 3.7 Analysis of cut-off values of differential metabolites between non-target metabolomics and lipidomics data groups

**Figure 5:**
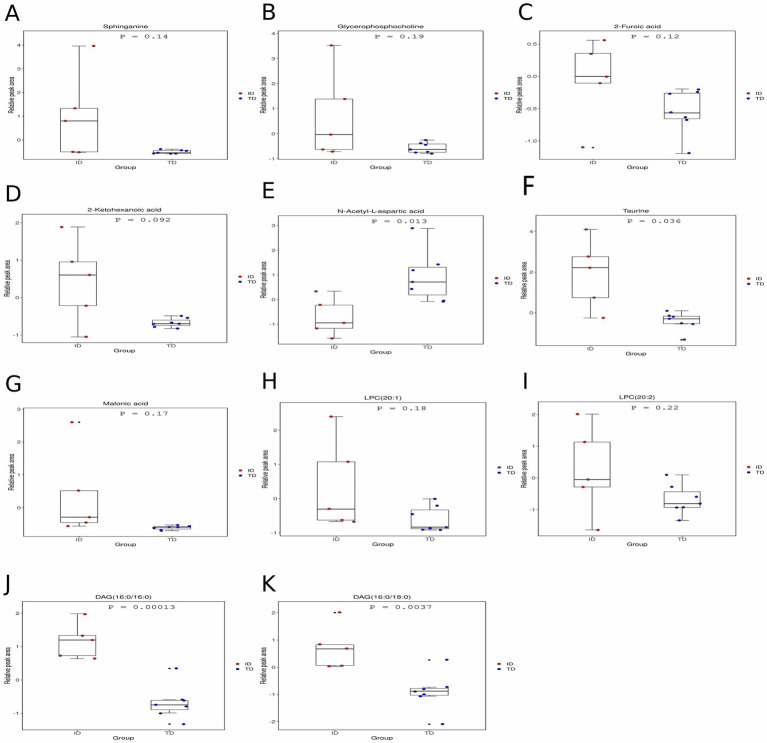
Relative peak area of candidate diagnostic markers. Scatter plots presenting levels of **(A)** sphinganine, **(B)** glycerophosphocholine, **(C)** 2-furoic acid, **(D)** 2-ketohexanoic acid, **(E)** *N*-acetyl-L-aspartic acid, **(F)** taurine, **(G)** malonic acid, **(H)** LPC (20:1), **(I)** LPC (20:2), **(J)** DAG (16:0/16:0), and **(K)** DAG (16:0/18:0) between GDD/ID and TD children.

Cut-off values of differential metabolites in the GDD/ID group were calculated using ROC curves and Youden’s index, as shown in Supplementary Table S5. Comprehensive diagnostic performance metrics for candidate biomarkers are summarized in Supplementary Table S5. Using cut-off values determined by Youden’s index, key biomarkers exhibited strong diagnostic efficacy: 2-ketohexanoic acid showed a sensitivity of 90.00% (95% confidence interval (CI): 73.48–98.79%) and specificity of 96.67% (95% CI: 82.78–99.92%); DAG (16:0/16:0) achieved a sensitivity of 93.30% (95% CI: 77.93–99.18%) and specificity of 86.70% (95% CI: 70.21–96.23%); glycerophosphocholine had a sensitivity of 73.30% (95% CI: 54.19–87.75%) and specificity of 100.00% (95% CI: 86.30–100.00%). Positive predictive values (PPV) and negative predictive values (NPV) for all biomarkers ranged from 75.00 to 100.00% and 66.67 to 96.15%, respectively, supporting their potential utility in clinical settings. For non-target metabolomics data, two positive ion mode differential metabolites were identified as potential biomarkers for GDD/ID, with AUCs of 0.899 and 0.859 for glycerophosphocholine and sphinganine, respectively. Five negative ion mode differential metabolites were identified as potential biomarkers for GDD/ID, with AUCs of 0.718, 0.912, 0.870, 0.789, and 0.861 for 2-furoic acid, 2-ketohexanoic acid, N-acetyl-L-aspartic acid, taurine, and malonic acid, respectively (Figures 5A–C, 4A–G). Four differential lipids were identified as potential biomarkers for GDD/ID, with AUCs of 0.753, 0.786, 0.956, and 0.949 for LPC (20:1), LPC (20:2), DAG (16:0/16:0), and DAG (16:0/18:0), respectively (Figures 5D, 4H–K).

## 4 Discussion

Existing omics technologies have great potential in monitoring the occurrence and development of diseases and clarifying biological processes, but so far, there has been no relevant research on metabolomics and lipidomics in GDD/ID. There are tens of thousands of metabolites in the human body, mainly including lipids, amino acids, organic acids, carbohydrates, and nucleic acids, with a variety of structures and significant differences in distribution and concentration in different tissues, cells, and body fluids. Numerous studies have found that metabolites that change significantly in nervous system diseases are lipids (Ferrer, 2018; Wood, 2014), indicating the importance of lipid metabolism disorders in neurodevelopmental conditions such as GDD/ID. Lipids are components of most bilayer cell membranes and play a crucial role in cell development, energy storage, signaling, material transport, apoptosis, immunity, angiogenesis, and inflammation (Zeng et al., 2017; Lu et al., 2021). Given the complexity of the etiology and pathogenesis of GDD/ID, the combination of metabolomics and lipidomics may be an important technology platform for discovering biomarkers of GDD/ID. Therefore, we combined metabolomics and lipidomics techniques to explore the profile of metabolites in the plasma of GDD/ID patients.

In this study, we explored differential metabolites in patients with GDD/ID and found 108 significantly altered small molecule metabolites, of which 48 lipid substances were identified as potential biomarkers. Most of the 108 differently metabolized small molecules belong to lipid and lipid-like categories, which is consistent with the conclusion that lipids are significantly altered in neurological diseases. Among the 48 different lipids, glycerol phospholipids and fatty acids constituted the majority. These differentially altered small molecule metabolites were mainly enriched in D-glutamine, D-glutamate, alanine, aspartic acid, lysine degradation, sphingolipid biosynthesis, linoleic acid metabolism, taurine, and the citric acid cycle. In contrast, the differentially altered lipids were primarily enriched in glycerol phospholipid and glycine metabolism. Using machine learning algorithms and ROC curve analysis, we explored potential biomarkers distinguishing GDD/ID from TD. Metabolite and lipid profiles in plasma samples could differentiate GDD/ID patients from TD. Eleven significantly altered metabolites and lipids were identified as potential biomarkers for GDD/ID. The AUC values for 2-ketohexanoic acid, DAG (16:0/16:0), and DAG (16:0/18:0) were greater than 0.9, indicating excellent diagnostic performance for GDD/ID. The AUC values of the other eight significantly altered metabolites and lipids ranged from 0.718 to 0.899, including glycerophosphocholine, sphinganine, 2-furoic acid, N-acetyl-L-aspartic acid, taurine, malonic acid, LPC (20:1), and LPC (20:2).

Long-term potentiation is well-established as playing a key role in synaptic plasticity, with NMDA receptors involved in the induction and regulation of both long-term potentiation and long-term inhibition in the hippocampus—processes critical for memory formation (Anwyl, 1999). NMDA receptors are localized to the postsynaptic membrane and exhibit a high affinity for glutamate (Uno and Coyle, 2019). Prior studies have suggested that disturbed glutamate metabolism may interfere with long-term potentiation and synaptic remodeling, potentially contributing to cognitive dysfunction (Christian et al., 2012). A key finding of this study is that glutamate, glutamine, and alanine metabolism are dysregulated in GDD/ID patients. This observation aligns with the known biological context: the glutamine–glutamate metabolic pathway connects neurons and glial cells in the central nervous system, maintaining the metabolic homeostasis of the excitatory neurotransmitter glutamate and clearing it from the synaptic cleft to prevent neuronal excitotoxicity (Dos Santos Quaresma et al., 2020; Tapiero et al., 2002). Based on this established pathway, we propose the hypothesis that dysregulation of glutamine and glutamate metabolism in GDD/ID patients may lead to abnormal release or transport clearance of glutamate in the synaptic cleft, potentially affecting interneuronal information transmission in the central nervous system and contributing to functional impairments, including developmental and intellectual deficits (Liu et al., 2022). However, this causal chain remains unvalidated in the current study.

Lysine is an essential amino acid for humans, and prior research has linked lysine deficiency to brain hypoplasia. Its most common degradation pathway is the saccharopine pathway. In studies of *Caenorhabditis elegans*, researchers found that abnormal function of saccharopine dehydrogenase in the lysine metabolic pathway leads to the accumulation of the intermediate metabolite saccharopine. This metabolite has been shown to disrupt the balance of mitochondrial division and fusion, delay mitophagy, and thereby cause mitochondrial homeostasis disorders and functional damage, highlighting the established importance of lysine metabolism in maintaining mitochondrial homeostasis (Zhou et al., 2019; Leandro and Houten, 2019). Aminoacyl-tRNA synthetase plays a well-documented role in aminoacyl-tRNA biosynthesis, as it can accurately recognize amino acid substrates and ensure the fidelity of protein synthesis (Ibba and Soll, 2000). Additionally, some studies have proposed that the loss of catalytic activity of mitochondrial arginyl-tRNA synthetase may lead to mitochondrial dysfunction, which is associated with pontine cerebellar hypoplasia (Edvardson et al., 2007). Malic acid is an intermediate metabolite of the citric acid cycle and is involved in mitochondrial energy metabolism. In our previous study, elevated malic acid levels were detected in the urine of GDD/ID patients; the current study further identifies malic acid as a significantly altered metabolite in the plasma of GDD/ID patients. This consistent finding supports the objective observation of abnormal tricarboxylic acid cycle metabolism or decreased malate–aspartate shuttle activity in GDD/ID patients. We tentatively hypothesize that this metabolic abnormality may be associated with disorders in neuronal mitochondrial energy metabolism and neurological dysfunction, but no causal relationship can be inferred from the current observational data.

Interestingly, metabolomic analysis revealed that GDD/ID patients exhibit not only dysregulation of amino acids, organic acids, and other metabolites but also significant alterations in lipid metabolism processes. Therefore, we performed a comprehensive quantitative lipidomic analysis on the plasma of GDD/ID patients, and an objective result is that differential lipids are mainly enriched in glycerophospholipid metabolism pathways. Glycerophospholipids are known to be key components of mitochondrial membrane structure, play important roles in cell signal transduction, and ensure the stability, fluidity, and permeability of neural membranes (Henry et al., 2012). They have also been reported to contribute to the regulation of liver lipid metabolism, memory promotion, immunity enhancement, and aging delay (Liu et al., 2022), and changes in glycerophospholipids in neural membranes have been associated with neurological diseases (Falabella et al., 2021; Ziegler and Tavosanis, 2019). Given that glycerophospholipids are the most abundant component of myelin in the meninges (van Meer et al., 2008), we propose the speculative hypothesis that glycerophospholipid metabolic dysregulation may be involved in the pathophysiological processes of GDD/ID, though this requires further mechanistic validation. Additionally, the endoplasmic reticulum is primarily composed of lipids and serves as an important organelle for protein and lipid synthesis, the removal of lipid-soluble metabolic wastes and harmful substances, and maintenance of calcium homeostasis. It is responsible for preserving fatty acid and lipid metabolic homeostasis, while lipid metabolism disorders have been shown to interfere with normal endoplasmic reticulum function. This disruption may further affect cell membrane structure and the functions of basic organelles such as mitochondria and the Golgi apparatus, thereby triggering endoplasmic reticulum stress and PERK pathway activation, which prior studies suggest may ultimately be associated with memory function impairment (Boggs, 2006).

Sphingolipid metabolism is well-recognized as crucial for maintaining the integrity and function of the nervous system (van Echten-Deckert and Alam, 2018; Olsen and Færgeman, 2017). Ceramide is the core molecule in sphingolipid metabolism, and both ceramide and other sphingolipids are known to play roles in learning and memory. Dysregulation of sphingolipid metabolism has been linked to impaired cellular function, various neuropsychiatric diseases, and accompanying cognitive dysfunction (Kalinichenko et al., 2022). Sphingosine, a degradation product of ceramide, is abundant in the central nervous system and plays established roles in brain development as well as the regulation of neuronal proliferation, differentiation, survival, and apoptosis (Pyne et al., 2016). Studies have shown significantly increased sphingosine levels in the serum of patients with autism spectrum disorder (ASD) (Wang et al., 2016). Furthermore, other researchers have demonstrated that treatment with FTY720, which reduces sphingosine-1-phosphate and sphingosine-1-phosphate receptor 1 levels, effectively restores learning and memory abilities in ASD model mice, increases the expression of cognition-related proteins in the hippocampus, and alleviates oxidative stress and neuronal loss (Wu et al., 2017). Additionally, reducing the expression of sphingosine-1-phosphate receptor 1 protein in a white matter injury model has been shown to improve white matter structure and enhance cognitive function (Serdar et al., 2016). These studies confirm the well-explored correlation between sphingosine and cognitive function, providing a basis for our tentative exploration of the potential mechanisms underlying GDD/ID.

Importantly, the current study is observational, and no causal relationships can be established between the identified metabolic perturbations and GDD/ID pathogenesis. All mechanistic links discussed above are tentative hypotheses derived from the correlation between metabolic profiles and clinical phenotypes, as well as alignment with prior literature. Correlation does not imply causation, and direct mechanistic experiments (e.g., *in vitro* neuronal models and *in vivo* animal studies) are needed to validate these hypotheses and clarify the direction of potential effects.

This study used two different algorithms to identify plasma markers potentially associated with GDD/ID. Seven significantly altered small molecule metabolites and four differential lipid substances were identified as potential biomarkers of GDD/ID. GDD/ID patients with identified differential metabolites or lipids can be treated early by supplementing the deficient metabolites, expelling the excess metabolites from their bodies, and avoiding contraindicated foods or drugs. The combined plasma metabonomics and lipidomics for the early identification of GDD/ID etiology can reveal differential metabolites and lipids, which might be valuable for future research on the etiology, pathogenesis, prognosis, and possible interventions for GDD/ID.

The intended clinical application of the identified biomarkers is early screening for GDD/ID, rather than definitive diagnosis. This distinction is critical: GDD/ID diagnosis requires comprehensive clinical assessment, including standardized developmental scales, neuroimaging, and genetic testing to rule out acquired or structural causes. However, the strong discriminative ability of our biomarkers (AUC > 0.85 for key metabolites/lipids) makes them valuable screening tools in primary care or pediatric settings. For example, in populations with high clinical suspicion (e.g., family history of neurodevelopmental disorders, subtle developmental delays), these biomarkers can help identify high-risk children who would benefit from timely referral for confirmatory diagnosis. Notably, the high NPV of top biomarkers (e.g., DAG (16:0/16:0) NPV = 93.5%) suggests they may also help rule out GDD/ID in low-risk individuals, reducing unnecessary diagnostic evaluations. It is important to emphasize that biomarkers alone cannot replace clinical judgment; rather, they serve as adjunctive tools to enhance early identification.

Global developmental delay (GDD) and intellectual disability (ID) are clinically heterogeneous, with variations in age at onset, trajectory, and severity—factors that may influence metabolic profiles. Given this, we performed post-hoc sensitivity analyses of 11 candidate biomarkers across age (≤3 vs. >3 years) and diagnosis (GDD-only vs. ID-only) subgroups. All biomarkers remained significantly dysregulated (*p* < 0.05) in both age subgroups, with AUC values (0.72–0.97) consistent with the total cohort, indicating they reflect core disease-specific processes rather than developmental fluctuations. Nine biomarkers were also dysregulated in both GDD-only and ID-only subgroups, confirming shared metabolic signatures linked to common pathogenic mechanisms (e.g., mitochondrial dysfunction, membrane abnormalities), while taurine and LPC (20:2) showed stronger diagnostic performance in ID-only (AUC = 0.92) than GDD-only (AUC = 0.75)—a trend potentially attributed to chronic neuronal dysfunction in ID, though limited by the small ID subgroup size (*n* = 8). While severe GDD/ID cases may involve more profound metabolic dysregulation, small sample size precluded formal severity-based analysis. Overall, these findings validate grouping GDD/ID for exploratory biomarker discovery, as core metabolic signatures are stable across subgroups while highlighting subgroup-specific trends worthy of large-scale validation.

Glutamate metabolism was dysregulated in GDD/ID patients. Several factors are known to influence plasma metabolomic and lipidomic profiles, including nutritional status, fasting conditions, and pharmacologic exposures. To minimize these confounders, we implemented strict standardized protocols: all participants fasted for 8–12 h prior to sampling, had normal BMI z-scores, and no history of chronic medication or supplement use in the 3 months before enrollment. These measures ensured that metabolic differences between groups were more likely attributable to GDD/ID itself rather than exogenous or nutritional factors. However, residual confounding cannot be completely excluded—for example, unmeasured dietary details (e.g., macronutrient intake ratios, food frequency) or short-term fluctuations in nutrient levels may have subtle effects on metabolic profiles. Future studies could address this by collecting detailed 24-h dietary recalls or food frequency questionnaires to adjust for nutritional variability more precisely. Additionally, while we excluded participants with chronic medication use, acute medication use (e.g., antibiotics within 1 week of sampling) was not systematically assessed, which could be considered in future study designs.

Despite the novel insights gained from this integrated metabolomic and lipidomic analysis of unexplained GDD/ID, several limitations should be acknowledged. First, this study was a single-center exploratory investigation with a modest sample size (30 GDD/ID cases and 30 TD controls), which is relatively small compared to the high dimensionality of metabolomic and lipidomic data. Although rigorous internal validation (200 permutation tests, cross-validation via SVM-RFE and LASSO algorithms) and post-hoc power analysis (achieving >80% power for key biomarkers) mitigated the risk of overfitting, the limited sample size may restrict the generalizability of our findings and increase the potential for false-positive associations. Second, the absence of external validation in independent cohorts—particularly those with diverse ethnic backgrounds, geographic distributions, and clinical severities—represents a critical gap. External validation is essential to confirm the diagnostic robustness and clinical utility of the identified biomarkers, as metabolomic profiles can be influenced by population-specific factors. Another key limitation is the lack of correlation between biomarker levels and clinical or developmental outcomes. We did not assess whether metabolite or lipid levels are associated with developmental quotient (DQ), adaptive functioning scores, or disease severity (mild/moderate/severe). This prevents us from determining whether the identified biomarkers can predict disease progression, response to intervention, or long-term outcomes—critical for enhancing their clinical utility. Future studies should collect detailed outcome data and explore these correlations to validate the prognostic value of the biomarkers. Third, while we standardized key confounding variables (e.g., fasting status, nutritional status, medication use) during recruitment, residual unmeasured confounders (e.g., detailed dietary patterns and environmental exposures) may have impacted the results.

To address these limitations and build on the current findings, future research should employ large-scale, multi-center designs with larger and more heterogeneous cohorts to validate the candidate biomarkers. Additionally, integrating multi-omics data (e.g., genomics, transcriptomics) with metabolomic and lipidomic profiles will provide a more comprehensive understanding of the molecular networks underlying GDD/ID. Rigorous experimental validation is also essential: *in vitro* studies using neuronal cell models (e.g., induced pluripotent stem cell-derived neurons from GDD/ID patients) will help clarify whether the identified metabolites and lipids (e.g., DAG (16:0/16:0), 2-ketohexanoic acid) directly regulate key neurodevelopmental processes such as synaptic plasticity, mitochondrial function, or lipid membrane homeostasis. *In vivo* studies using animal models of GDD/ID (e.g., mice with genetic mutations linked to metabolic dysregulation) will enable assessment of the causal role of these metabolic perturbations in disease pathogenesis, as well as the potential therapeutic effects of targeting these pathways. Orthogonal validation of the 11 putative biomarkers using enzyme-linked immunosorbent assay (ELISA) in an expanded, independent cohort is also planned to confirm their reliability beyond computational analysis. Collectively, these future studies will strengthen the clinical relevance of our findings, elucidate the causal relationships between the identified metabolic perturbations and GDD/ID pathogenesis, and advance the development of novel screening and targeted intervention strategies for GDD/ID.

## Data Availability

The original contributions presented in the study are included in the article/supplementary material, further inquiries can be directed to the corresponding author.

## Footnotes

1
https://www.metaboanalyst.ca/

## Supplementary material

Supplementary Figure S1 Analysis of metabolomics and lipidomics of GDD/ID and TD groups. **(A,D)** Score scatter plot of PCA and OPLS-DA model for positive ion mode of non-target metabolomics data. **(B,E)** Score scatter plot of PCA and OPLS-DA model for negative ion mode of non-target metabolomics data. **(C,F)** Score scatter plot of PCA and OPLS-DA model for lipidomics data. **(G)** Permutation test of OPLS-DA model for positive ion mode of non-target metabolomics data. **(H)** Permutation test of OPLS-DA model for negative ion mode of non-target metabolomics data. **(I)** permutation test of OPLS-DA model for lipidomics data.

Supplementary Figure S2 Metabolomics and lipidomics differential metabolites between GDD/ID and TD groups. **(A,B)** Volcano plots showing differences in positive ion mode **(A)** and negative ion mode **(B)** of metabolites and lipids **(C)** between GDD/ID patients and TD children: upregulated metabolites (red circles), downregulated metabolites (blue circles). The numbers and proportions of differential polar metabolites for positive ion mode **(D)**, negative ion mode **(E)**, and differential polar lipids **(F)**.

Supplementary Figure S3 Correlation analysis of differential metabolites between non-target metabolomics data and lipidomics groups. Heatmap of correlation analysis in positive ion mode **(A)** and negative ion mode **(B)** of metabolites, and lipids **(C)** between GDD/ID patients and TD children. The horizontal and vertical coordinates represent the differential metabolites in the comparison group, and the color blocks at different positions represent the correlation coefficients between metabolites at corresponding positions. Red represents the positive correlation, blue represents the negative correlation, and the darker the color, the stronger the correlation.

## References

[ref1] Anwyl R. (1999). Metabotropic glutamate receptors: electrophysiological properties and role in plasticity. Brain Res. Brain Res. Rev. 29, 83–120. doi: 10.1016/S0165-0173(98)00050-2, 9974152

[ref2] Bélanger S. A. Caron J. (2018). Evaluation of the child with global developmental delay and intellectual disability. Paediatr. Child Health 23, 403–419. doi: 10.1093/pch/pxy093, 30919832 PMC6234423

[ref3] Berger J. Dorninger F. Forss-Petter S. Kunze M. (2016). Peroxisomes in brain development and function. Biochim. Biophys. Acta 1863, 934–955. doi: 10.1016/j.bbamcr.2015.12.005, 26686055 PMC4880039

[ref4] Boggs J. M. (2006). Myelin basic protein: a multifunctional protein. Cell. Mol. Life Sci. 63, 1945–1961. doi: 10.1007/s00018-006-6094-7, 16794783 PMC11136439

[ref5] Boustany R. M. (2013). Lysosomal storage diseases--the horizon expands. Nat. Rev. Neurol. 9, 583–598. doi: 10.1038/nrneurol.2013.163, 23938739

[ref6] Burrage L. C. Nagamani S. C. Campeau P. M. Lee B. H. (2014). Branched-chain amino acid metabolism: from rare Mendelian diseases to more common disorders. Hum. Mol. Genet. 23, R1–R8. doi: 10.1093/hmg/ddu123, 24651065 PMC4170715

[ref7] Chen X. Lee J. Wu H. Tsang A. W. Furdui C. M. (2019). Mass spectrometry in advancement of redox precision medicine. Adv. Exp. Med. Biol. 1140, 327–358. doi: 10.1007/978-3-030-15950-4_19, 31347057 PMC9236553

[ref8] Chen Q. Qiao Y. Xu X. J. You X. Tao Y. (2019). Urine organic acids as potential biomarkers for autism-spectrum disorder in Chinese children. Front. Cell. Neurosci. 13:150. doi: 10.3389/fncel.2019.00150, 31114480 PMC6502994

[ref9] Christian D. T. Alexander N. J. Diaz M. R. Robinson S. McCool B. A. (2012). Chronic intermittent ethanol and withdrawal differentially modulate basolateral amygdala AMPA-type glutamate receptor function and trafficking. Neuropharmacology 62, 2430–2439. doi: 10.1016/j.neuropharm.2012.02.017, 22387532 PMC3314101

[ref10] Daci A. Bozalija A. Jashari F. Krasniqi S. (2018). Individualizing treatment approaches for epileptic patients with glucose transporter type1 (GLUT-1) deficiency. Int. J. Mol. Sci. 19:122. doi: 10.3390/ijms19010122, 29303961 PMC5796071

[ref11] DeBarber A. E. Eroglu Y. Merkens L. S. Pappu A. S. Steiner R. D. (2011). Smith-Lemli-Opitz syndrome. Expert Rev. Mol. Med. 13:e24. doi: 10.1017/S146239941100189X, 21777499 PMC3366105

[ref12] Dos Santos Quaresma M. Souza W. Lemos V. A. Caris A. V. Thomatieli-Santos R. V. (2020). The possible importance of glutamine supplementation to mood and cognition in hypoxia from high altitude. Nutrients 12:3627. doi: 10.3390/nu12123627, 33255790 PMC7760805

[ref13] Edvardson S. Shaag A. Kolesnikova O. Gomori J. M. Tarassov I. Einbinder T. . (2007). Deleterious mutation in the mitochondrial arginyl-transfer RNA synthetase gene is associated with pontocerebellar hypoplasia. Am. J. Hum. Genet. 81, 857–862. doi: 10.1086/521227, 17847012 PMC2227936

[ref14] Falabella M. Vernon H. J. Hanna M. G. Claypool S. M. Pitceathly R. D. S. (2021). Cardiolipin, mitochondria, and neurological disease. Trends Endocrinol. Metab. 32, 224–237. doi: 10.1016/j.tem.2021.01.006, 33640250 PMC8277580

[ref15] Ferrer I. (2018). Proteomics and lipidomics in the human brain. Handb. Clin. Neurol. 150, 285–302. doi: 10.1016/B978-0-444-63639-3.00020-7, 29496147

[ref16] Flippo K. H. Strack S. (2017). Mitochondrial dynamics in neuronal injury, development and plasticity. J. Cell Sci. 130, 671–681. doi: 10.1242/jcs.171017, 28154157 PMC5339882

[ref17] Gabbi P. Ribeiro L. R. Jessié Martins G. Cardoso A. S. Haupental F. Rodrigues F. S. . (2017). Methylmalonate induces inflammatory and apoptotic potential: a link to glial activation and neurological dysfunction. J. Neuropathol. Exp. Neurol. 76, 160–178. doi: 10.1093/jnen/nlw121, 28395089

[ref18] García-Cazorla A. Wolf N. I. Serrano M. Moog U. Pérez-Dueñas B. Póo P. . (2009). Mental retardation and inborn errors of metabolism. J. Inherit. Metab. Dis. 32, 597–608. doi: 10.1007/s10545-009-0922-5, 19685154

[ref19] Han X. Yang K. Gross R. W. (2012). Multi-dimensional mass spectrometry-based shotgun lipidomics and novel strategies for lipidomic analyses. Mass Spectrom. Rev. 31, 134–178. doi: 10.1002/mas.20342, 21755525 PMC3259006

[ref20] Henry S. A. Kohlwein S. D. Carman G. M. (2012). Metabolism and regulation of glycerolipids in the yeast *Saccharomyces cerevisiae*. Genetics 190, 317–349. doi: 10.1534/genetics.111.130286, 22345606 PMC3276621

[ref21] Ibba M. Soll D. (2000). Aminoacyl-tRNA synthesis. Annu. Rev. Biochem. 69, 617–650. doi: 10.1146/annurev.biochem.69.1.617, 10966471

[ref22] Kalinichenko L. S. Gulbins E. Kornhuber J. Müller C. P. (2022). Sphingolipid control of cognitive functions in health and disease. Prog. Lipid Res. 86:101162. doi: 10.1016/j.plipres.2022.101162, 35318099

[ref23] Leandro J. Houten S. M. (2019). Saccharopine, a lysine degradation intermediate, is a mitochondrial toxin. J. Cell Biol. 218, 391–392. doi: 10.1083/jcb.201901033, 30651301 PMC6363453

[ref24] Lichter-Konecki U. Vockley J. (2019). Phenylketonuria: current treatments and future developments. Drugs 79, 495–500. doi: 10.1007/s40265-019-01079-z, 30864096

[ref25] Lim Y. T. Mankad K. Kinali M. Tan A. P. (2020). Neuroimaging spectrum of inherited neurotransmitter disorders. Neuropediatrics 51, 6–21. doi: 10.1055/s-0039-1698422 31634934

[ref26] Liu D. Huang J. Gao S. Jin H. He J. (2022). A temporo-spatial pharmacometabolomics method to characterize pharmacokinetics and pharmacodynamics in the brain microregions by using ambient mass spectrometry imaging. Acta Pharm. Sin. B 12, 3341–3353. doi: 10.1016/j.apsb.2022.03.018, 35967273 PMC9366215

[ref27] Lu J. Guo Y. Lu Y. Ji W. Lin L. Qian W. . (2021). Untargeted lipidomics reveals specific lipid abnormalities in Sjögren's syndrome. Rheumatology (Oxford) 60, 1252–1259. doi: 10.1093/rheumatology/keaa456, 32911538

[ref28] Matsumoto S. Häberle J. Kido J. Mitsubuchi H. Endo F. Nakamura K. (2019). Urea cycle disorders-update. J. Hum. Genet. 64, 833–847. doi: 10.1038/s10038-019-0614-4, 31110235

[ref29] Olsen A. S. B. Færgeman N. J. (2017). Sphingolipids: membrane microdomains in brain development, function and neurological diseases. Open Biol. 7:170069. doi: 10.1098/rsob.170069, 28566300 PMC5451547

[ref30] Pyne S. Adams D. R. Pyne N. J. (2016). Sphingosine 1-phosphate and sphingosine kinases in health and disease: recent advances. Prog. Lipid Res. 62, 93–106. doi: 10.1016/j.plipres.2016.03.001, 26970273

[ref31] Reily C. Stewart T. J. Renfrow M. B. Novak J. (2019). Glycosylation in health and disease. Nat. Rev. Nephrol. 15, 346–366. doi: 10.1038/s41581-019-0129-4, 30858582 PMC6590709

[ref32] Serdar M. Herz J. Kempe K. Lumpe K. Reinboth B. S. Sizonenko S. V. . (2016). Fingolimod protects against neonatal white matter damage and long-term cognitive deficits caused by hyperoxia. Brain Behav. Immun. 52, 106–119. doi: 10.1016/j.bbi.2015.10.004, 26456693

[ref33] Sharer J. D. Bodamer O. Longo N. Tortorelli S. Wamelink M. M. Young S. (2017). Laboratory diagnosis of creatine deficiency syndromes: a technical standard and guideline of the American College of Medical Genetics and Genomics. Genet. Med. 19, 256–263. doi: 10.1038/gim.2016.203, 28055022

[ref34] Tapiero H. Mathé G. Couvreur P. Tew K. D. (2002). II. Glutamine and glutamate. Biomed. Pharmacother. 56, 446–457. doi: 10.1016/S0753-3322(02)00285-8, 12481981

[ref35] Uno Y. Coyle J. T. (2019). Glutamate hypothesis in schizophrenia. Psychiatry Clin. Neurosci. 73, 204–215. doi: 10.1111/pcn.12823, 30666759

[ref36] van Echten-Deckert G. Alam S. (2018). Sphingolipid metabolism - an ambiguous regulator of autophagy in the brain. Biol. Chem. 399, 837–850. doi: 10.1515/hsz-2018-0237, 29908127

[ref37] van Hoytema Konijnenburg E. M. M. Wortmann S. B. Koelewijn M. J. Tseng L. A. Houben R. Stöckler-Ipsiroglu S. . (2021). Treatable inherited metabolic disorders causing intellectual disability: 2021 review and digital app. Orphanet J. Rare Dis. 16:170. doi: 10.1186/s13023-021-01727-2, 33845862 PMC8042729

[ref38] van Karnebeek C. D. (2014). Inborn errors of metabolism are not hopeless; early identification of treatable conditions in children with intellectual disability. Ned. Tijdschr. Geneeskd. 158:A8042. 25424630

[ref39] van Meer G. Voelker D. R. Feigenson G. W. (2008). Membrane lipids: where they are and how they behave. Nat. Rev. Mol. Cell Biol. 9, 112–124. doi: 10.1038/nrm2330, 18216768 PMC2642958

[ref40] Vasudevan P. Suri M. (2017). A clinical approach to developmental delay and intellectual disability. Clin. Med. (Lond.) 17, 558–561. doi: 10.7861/clinmedicine.17-6-558, 29196358 PMC6297696

[ref41] Wang H. Liang S. Wang M. Gao J. Sun C. Wang J. . (2016). Potential serum biomarkers from a metabolomics study of autism. J. Psychiatry Neurosci. 41, 27–37. doi: 10.1503/jpn.140009, 26395811 PMC4688025

[ref42] Wishart D. S. (2016). Emerging applications of metabolomics in drug discovery and precision medicine. Nat. Rev. Drug Discov. 15, 473–484. doi: 10.1038/nrd.2016.32, 26965202

[ref43] Wood P. L. (2014). Mass spectrometry strategies for clinical metabolomics and lipidomics in psychiatry, neurology, and neuro-oncology. Neuropsychopharmacology 39, 24–33. doi: 10.1038/npp.2013.167, 23842599 PMC3857645

[ref44] Wu H. Wang X. Gao J. Liang S. Hao Y. Sun C. . (2017). Fingolimod (FTY720) attenuates social deficits, learning and memory impairments, neuronal loss and neuroinflammation in the rat model of autism. Life Sci. 173, 43–54. doi: 10.1016/j.lfs.2017.01.012, 28161158

[ref45] Yoo J. S. Ryu C. H. Kim Y. S. Kim H. J. Bushnell C. D. Kim H. Y. (2020). Homocysteinemia is associated with the presence of microbleeds in cognitively impaired patients. J. Stroke Cerebrovasc. Dis. 29:105302. doi: 10.1016/j.jstrokecerebrovasdis.2020.105302, 32992197

[ref46] Zeng C. Wen B. Hou G. Lei L. Mei Z. Jia X. . (2017). Lipidomics profiling reveals the role of glycerophospholipid metabolism in psoriasis. Gigascience 6, 1–11. doi: 10.1093/gigascience/gix087 PMC5647792 29046044

[ref47] Zhou J. Wang X. Wang M. Chang Y. Zhang F. Ban Z. . (2019). The lysine catabolite saccharopine impairs development by disrupting mitochondrial homeostasis. J. Cell Biol. 218, 580–597. doi: 10.1083/jcb.201807204, 30573525 PMC6363459

[ref48] Ziegler A. B. Tavosanis G. (2019). Glycerophospholipids - emerging players in neuronal dendrite branching and outgrowth. Dev. Biol. 451, 25–34. doi: 10.1016/j.ydbio.2018.12.009, 30576627

[ref49] Züllig T. Trötzmüller M. Köfeler H. C. (2020). Lipidomics from sample preparation to data analysis: a primer. Anal. Bioanal. Chem. 412, 2191–2209. doi: 10.1007/s00216-019-02241-y, 31820027 PMC7118050

