# Supplementary figures and images for: The integration of plasma non-target metabolomics and lipidomics analysis for the discovery of global developmental delay/intellectual disability biomarkers
Source: Front Cell Neurosci. 2026 Feb 4;20:1688339. doi: 10.3389/fncel.2026.1688339 (PMC12913114; doi:10.3389/fncel.2026.1688339)

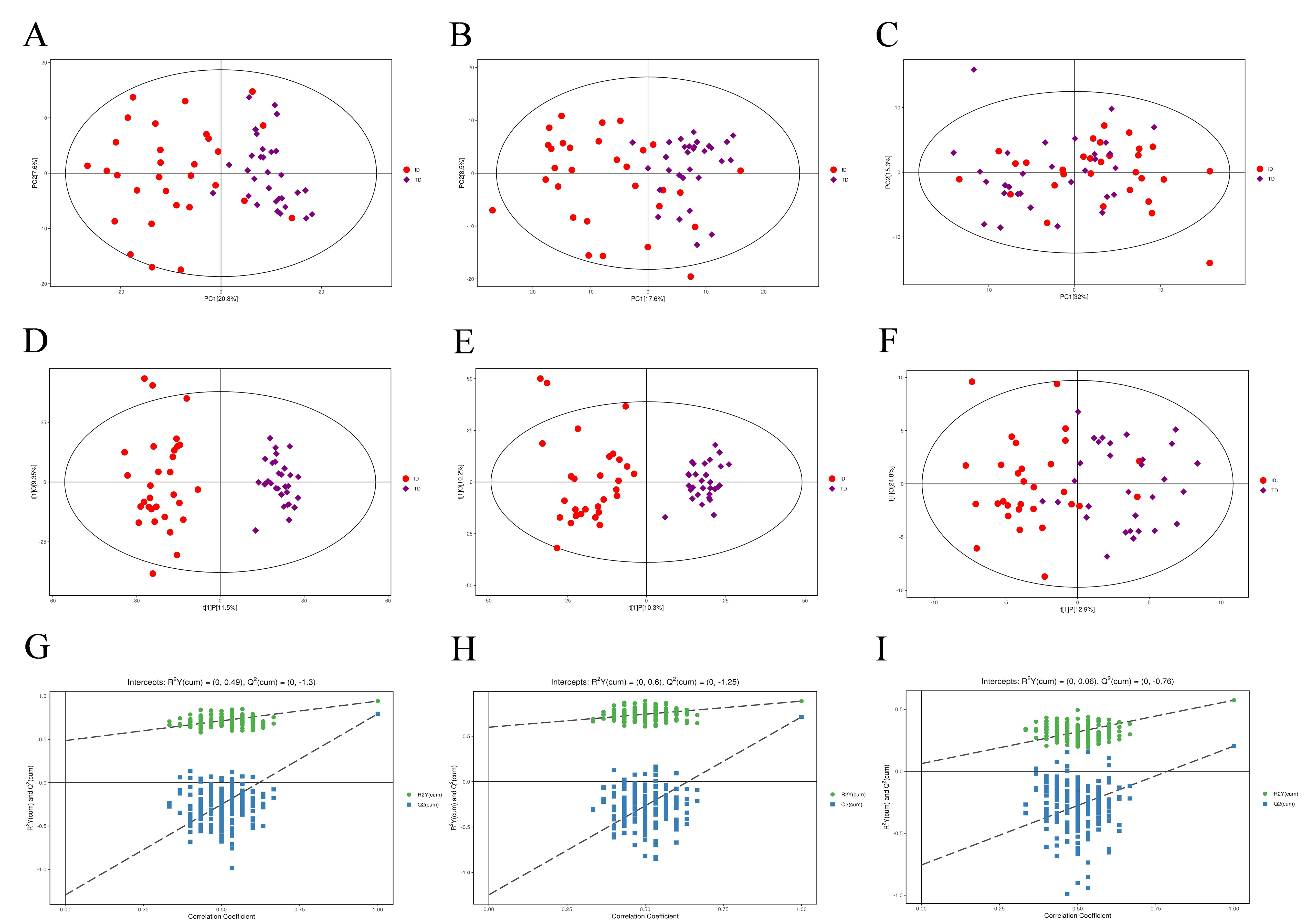

Supplement: Supplementary Figure S1 — Analysis of metabolomics and lipidomics of GDD/ID and TD groups. (A,D) Score scatter plot of PCA and OPLS-DA model for positive ion mode of non-target metabolomics data. (B,E) Score scatter plot of PCA and OPLS-DA model for negative ion mode of non-target metabolomics data. (C,F) Score scatter plot of PCA and OPLS-DA model for lipidomics data. (G) Permutation test of OPLS-DA model for positive ion mode of non-target metabolomics data. (H) Permutation test of OPLS-DA model for negative ion mode of non-target metabolomics data. (I) permutation test of OPLS-DA model for lipidomics data. [file Image_1.JPEG]

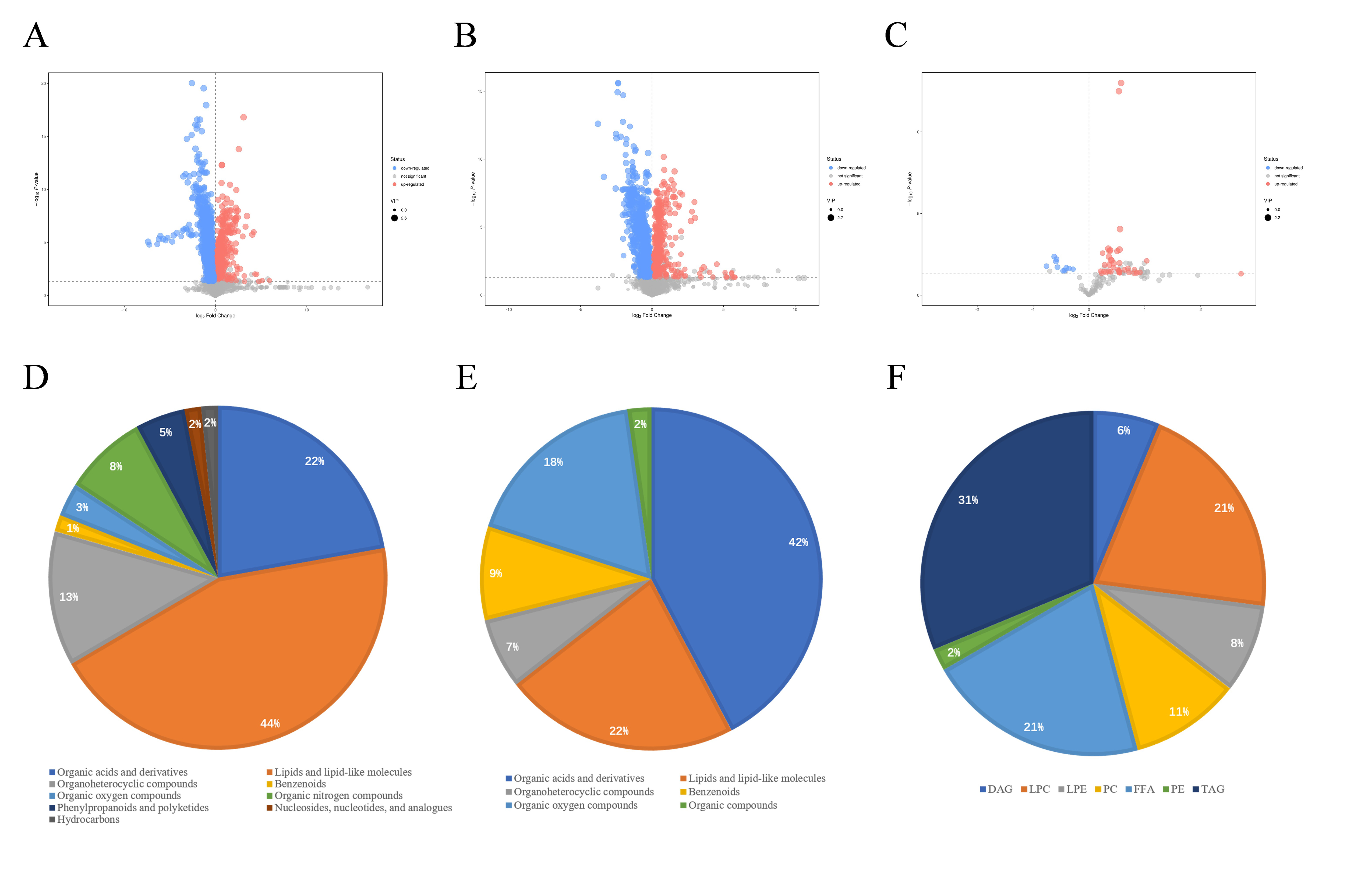

Supplement: Supplementary Figure S2 — Metabolomics and lipidomics differential metabolites between GDD/ID and TD groups. (A,B) Volcano plots showing differences in positive ion mode (A) and negative ion mode (B) of metabolites and lipids (C) between GDD/ID patients and TD children: upregulated metabolites (red circles), downregulated metabolites (blue circles). The numbers and proportions of differential polar metabolites for positive ion mode (D), negative ion mode (E), and differential polar lipids (F). [file Image_2.JPEG]

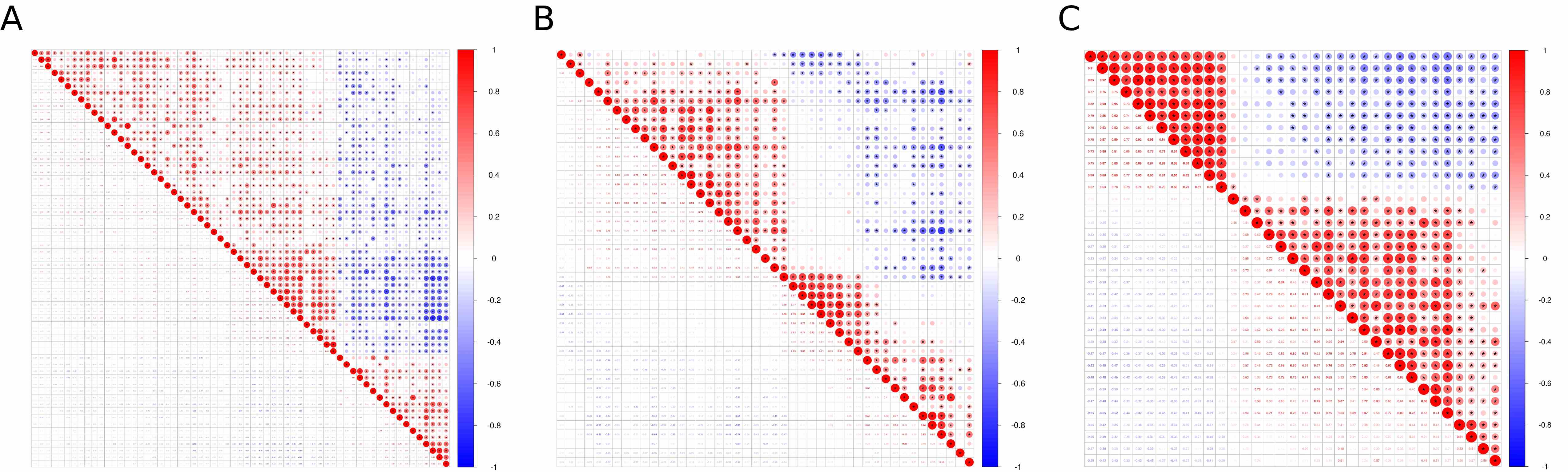

Supplement: Supplementary Figure S3 — Correlation analysis of differential metabolites between non-target metabolomics data and lipidomics groups. Heatmap of correlation analysis in positive ion mode (A) and negative ion mode (B) of metabolites, and lipids (C) between GDD/ID patients and TD children. The horizontal and vertical coordinates represent the differential metabolites in the comparison group, and the color blocks at different positions represent the correlation coefficients between metabolites at corresponding positions. Red represents the positive correlation, blue represents the negative correlation, and the darker the color, the stronger the correlation. [file Image_3.JPEG]
