# Supplementary material for: The integration of plasma non-target metabolomics and lipidomics analysis for the discovery of global developmental delay/intellectual disability biomarkers
Source: Front Cell Neurosci. 2026 Feb 4;20:1688339. doi: 10.3389/fncel.2026.1688339 (PMC12913114; doi:10.3389/fncel.2026.1688339)
Supplement: Supplementary file 4 [file Table_1.DOCX]

**Table S1. Characteristics of the study population**

|  | GDD/ID group | TD group | *p*-value |
| --- | --- | --- | --- |
| Number of subjects | 30 | 30 |  |
| Age (mean, range) | 3.13 (1.79,5.04) | 4.54 (2.73,7.00) | 0.08 |
| Gender |  |  | 0.41 |
| Male | 22 (73.33%) | 18 (60.00%) |  |
| Female | 8 (26.67%) | 12 (40.00%) |  |
| Race | Chinese | Chinese |  |

**Table S2. Parameter table of PCA and OPLS-DA model for non-target metabolomics and lipidomics data**

| Omics methods | Type | Number of principal components of the model | Number of samples | R^2^X（cum） | R^2^Y（cum） | Q^2^（cum） |
| --- | --- | --- | --- | --- | --- | --- |
| Non-target metabolomics | PCA | 7 | 60 | 0.507 |  |  |
|  | OPLS-DA | 1+1+0 | 60 | 0.208 | 0.942 | 0.795 |
| Lipidomics | PCA | 3 | 60 | 0.555 |  |  |
|  | OPLS-DA | 1+1+0 | 60 | 0.377 | 0.574 | 0.204 |

**Table S5. Analysis of cut-off values of differential metabolites between non-target metabolomics data and lipidomics data groups**

| Differential metabolites | Cut-off Value | AUC | Sensitivity  (%) | Specificity  (%) | Youden index | *P -*value | 95% CI |
| --- | --- | --- | --- | --- | --- | --- | --- |
| Glycerophosphocholine | 0.206 | 0.899 | 73.30 | 100.00 | 0.733 | 0.000 | 0.821, 0.977 |
| Sphinganine | 0.006 | 0.859 | 60.00 | 100.00 | 0.600 | 0.000 | 0.768, 0.950 |
| 2-Furoic acid | 0.080 | 0.718 | 60.00 | 80.00 | 0.400 | 0.004 | 0.587, 0.848 |
| 2-Ketohexanoic acid | 0.285 | 0.912 | 90.00 | 96.67 | 0.867 | 0.000 | 0.819, 1.000 |
| N-Acetyl-L-aspartic acid | 0.202 | 0.870 | 80.00 | 90.00 | 0.700 | 0.000 | 0.777, 0.963 |
| Taurine | 6.126 | 0.789 | 53.33 | 96.67 | 0.500 | 0.000 | 0.672, 0.906 |
| Malonic acid | 0.637 | 0.861 | 70.00 | 93.33 | 0.633 | 0.000 | 0.767, 0.956 |
| LPC (20:1) | 185.285 | 0.753 | 66.70 | 80.00 | 0.467 | 0.001 | 0.628, 0.878 |
| LPC (20:2) | 104.711 | 0.786 | 83.30 | 76.70 | 0.600 | 0.000 | 0.663, 0.908 |
| DAG (16:0/16:0) | 2168.637 | 0.956 | 93.30 | 86.70 | 0.800 | 0.000 | 0.910, 1.000 |
| DAG (16:0/18:0) | 1094.447 | 0.949 | 96.70 | 86.70 | 0.833 | 0.000 | 0.896, 1.000 |
